# Supplementary material for: Identification of photoperiod-induced specific miRNAs in the adrenal glands of Sunite sheep (Ovis aries)
Source: Front Vet Sci. 2022 Jul 22;9:888207. doi: 10.3389/fvets.2022.888207 (PMC9354845; doi:10.3389/fvets.2022.888207)
Supplement: Supplementary file 1 [file Data_Sheet_1.ZIP › Supplementary file/miRDeep2 file.zip!/miRDeep2 file/Know_Structure/LP42A2/oar-mir-3959.pdf]

miRBase precursor : oar-mir-3959
Total read count : 16177
oar-miR-3959-5p read count : 13365
oar-miR-3959-3p read count : 2810
remaining reads : 2

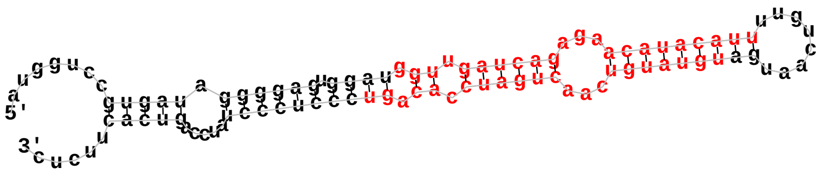

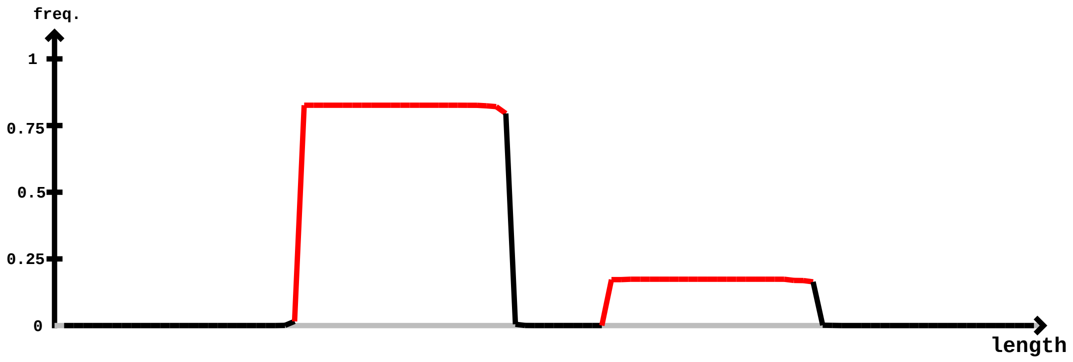

oar-miR-3959-5p

oar-miR-3959-3p

```
5'- augguccgugauaggggaguggaugguugaucagagaacauacauuuugucaaugauguaugucaacugauccacagucccucccuauccagucacuucuc -3'   exp
    .......(((((.((((((.((((.((.((((((...(((((((((........)))))))))...)))))).)).)))))))))).....))))).....      reads   mm   sample
    ......................augguugaucagagaacauac..........................................................      5       0    seq
    ......................augguugaucagagaacauaca.........................................................      7       0    seq
    .......................ugguugaucagagaacaua...........................................................      1       0    seq
    .......................ugguugaucagagaacauaca.........................................................      10      0    seq
    .......................ugguugaucagagaacauacau........................................................      15      0    seq
    .......................ugguugaucagagaacauacauu.......................................................      215     0    seq
    .......................ugguugaucagagaacauacauuu......................................................      1       0    seq
    ........................gguugaucagagaacau............................................................      2       0    seq
    ........................gguugaucagagaacaua...........................................................      2       0    seq
    ........................gguugaucagagaacauac..........................................................      27      0    seq
    ........................gguugaucagagaacauaca.........................................................      32      0    seq
    ........................gguugaucagagaacauacau........................................................      398     0    seq
    ........................gguugaucagagaacauacauu.......................................................      12574   0    seq
    ........................gguugaucagagaacauacauuu......................................................      71      0    seq
    ........................gguugaucagagaacauacauuuu.....................................................      5       0    seq
    ..............................................uugucaaugauguaugucaacugauccacagu.......................      2       0    seq
    ........................................................uguaugucaacugaucca...........................      2       0    seq
    ........................................................uguaugucaacugauccac..........................      72      0    seq
    ........................................................uguaugucaacugauccaca.........................      9       0    seq
    ........................................................uguaugucaacugauccacag........................      77      0    seq
    ........................................................uguaugucaacugauccacagu.......................      2619    0    seq
    ........................................................uguaugucaacugauccacaguc......................      6       0    seq
    .........................................................guaugucaacugauccacagu.......................      1       0    seq
    ..........................................................uaugucaacugauccacagu.......................      9       0    seq
    ..........................................................uaugucaacugauccacaguc......................      5       0    seq
    ..........................................................uaugucaacugauccacagucc.....................      10      0    seq
```
